# Analysis of influencing factors on the rate of high-quality embryos induced by rectangular plan during in vitro fertilization

**DOI:** 10.12669/pjms.41.4.10188

**Published:** 2025-04

**Authors:** Hongzhi Shi, Zhi-ming Zhao, Qi Song, Jiajia Liu

**Affiliations:** 1 Hongzhi Shi, Department of Reproductive Medicine, Maternity & Child Care Center of Qinhuangdao, Qinhuangdao, 066000, Hebei, China; 2 Zhi-ming Zhao, Department of Reproductive Medicine, The Second Hospital of Hebei Medical University, Shijiazhuang, Hebei, 050000, China; 3 Qi Song, Department of Reproductive Medicine, Maternity & Child Care Center of Qinhuangdao, Qinhuangdao, 066000, Hebei, China; 4 Jiajia Liu, Department of Reproductive Medicine, Maternity & Child Care Center of Qinhuangdao, Qinhuangdao, 066000, Hebei, China

**Keywords:** Blastocyst formation rate, High-quality embryo, In vitro fertilization, Long protocol, Progesterone on the day of hCG injection

## Abstract

**Objective:**

To explore factors influencing high-quality embryo rate in long protocols for controlled ovarian hyperstimulation (COH) during in vitro fertilization (IVF).

**Methods:**

It was a retrospective case-control study including patients receiving assisted pregnancy treatment at the Reproductive Medicine Department of the Maternal & Child Care Center of Qinhuangdao from January 2018 to October 2023. This study adopted 724 treatment cycles using COH involving intermediate-acting and long-acting protocols. According to the high-quality embryo rate on D3, the enrolled patients were divided into Group-A for control (high-quality embryo rate ≥40%, n=297) and Group-B without high-quality embryo (excellent embryo rate=0, n=427). Factors with P<0.05 in the univariate analysis were incorporated into the Logistic regression model for binary logistic regression analysis.

**Results:**

There were no statistically significant differences in male age, years of infertility, body mass index (BMI), baseline follicle-stimulating hormone (FSH) levels, AMH, total gonadotropin (Gn)dose, total days of Gn administration, sperm concentration, motility, normal morphology rate, and LH levels on the day of human chorionic gonadotropin (hCG) injection between Group-A and Group-B (all p>0.05). In addition, the Logistic binary regression analysis showed that in the cycle without high-quality embryos on D3, elevated progesterone on the day of hCG injection was an independent risk factor affecting the developmental potential of late-stage embryos.

**Conclusion:**

For cycles with poor embryo quality, the increase in progesterone levels in the late stage of COH has a significant negative impact on the formation of effective blastocysts.

## INTRODUCTION

During controlled ovarian hyperstimulation (COH) for in vitro fertilization (IVF), the use of long protocols can reduce the proportion of whole embryo freezing (“freeze-all”) due to its good synchronization between the development of the embryo and the differentiation of the endometriu^1^, leading to improved efficacy and stable success rate of fresh-cycle embryo transfer. During treatment using long protocols, great concern has hence been attached continuously to improving the quality of embryos in this cycle, i.e., improving the utilization efficiency of oocytes while achieving a certain number of retrieved oocytes, in the field of reproductive medicine.^2^,^3^

This study analyzed from multiple aspects such as the physical condition of female patients, male age, sperm quality, and COH process, and carried out subgroup-Analysis on the developmental potential of the group with poor embryo quality. Significantly, grouping based on the rate of effective blastocysts for freezing, using valuable embryos that can be used for freezing as the numerator and the number of embryos cultured as the denominator, is more intuitive for observing later-stage embryonic development than traditional blastocyst formation rate. This grouping method can reduce the interference of ineffective blastocysts, such as phase II blastocyst, where the inner cell mass and trophoblast cells are both grade CC inferior blastocysts, occurring more frequently during cycles of poor embryo quality. There may be biased data if the traditional blastocyst formation rate is used for calculation and analysis. It can better explore factors influencing high-quality embryo rate in long protocols for controlled ovarian hyperstimulation (COH) during in vitro fertilization (IVF).

## METHODS

A retrospective analysis was performed on the clinical data of patients undergoing in vitro fertilization-embryo transfer (IVF-ET) in Maternity & Child Care Center of Qinhuangdao from January 2018 to October 2023. Data were retrieved from the hospital information and management system, and collect their various information of all patients.

### Ethical Approval:

The study was approved by the Institutional Ethics Committee of Maternity & Child Care Center of Qinhuangdao (No. QHDFY-2023031008; Date: March 10, 2023) and written informed consent was obtained from all participants and their families.

### Inclusion criteria:

- Females with long-acting long protocols in the mid-luteal phase and early follicular phase.
- Females with ≥4 oocytes retrieved.
- Females aged 22-45 years old.
- Females with baseline follicle-stimulating hormone (FSH) <10U/L.

### Exclusion criteria:

- Females with endometriosis.
- Females with thyroid disorder.
- Females with diabetes and other diseases.

### COH regimen:

Long-acting long protocol in the early follicular phase: administration of long-acting GnRH-a (3.75 mg) on 2-4 days of the menstrual cycle for 30-40 days of downregulation. Long-acting long protocol in the mid-luteal phase: administration of long-acting GnRH-a (3.75 mg) during the mid-luteal phase of the previous menstrual cycle for 14-16 days of downregulation, with a purpose to achieve pituitary downregulation for COH. The dosage of Gn was adjusted during the medication based on ovarian response and hormone levels.

When the diameter was observed to be ≥20 mm in ≥1 dominant follicle(s), or ≥18 mm in three dominant follicles, gonadotropin (Gn) administration was withdrawn and recombinant human chorionic gonadotropin (hCG; 250 μg; Eiser, Merck Serono, Switzerland) was used for triggering ovulation, followed by oocyte retrieval 36-38 hours later. Routine IVF/ICSI was performed subsequently according to the semen of the male partner and the patient’s condition.

Semen processing and fertilization: Before sperm retrieval, the male partner was required to abstain from sexual activity for 2-7 days, and collect sperm through masturbation on the day of sperm collection. The semen was processed by using density gradient centrifugation combined with swim-up method. The separation medium for density gradient centrifugation used 40% Pureception and 80% Pureception (Cooper, USA), and the washing-up liquid and swim-up liquid were respectively the sperm and fertilization medium (ART-1020, Sage, USA). Fertilization occurred 38-40 h after hCG injection.

### Embryo observation:

Embryo formation was observed on D3 after fertilization. The evaluation criteria for the quality of cleavage-stage embryos can be found in the reference.^4^ The blastocyst quality was evaluated based on the Gardner scoring system.^5^

### Grouping of the study:

According to the high-quality embryo rate on D3, the enrolled patients were divided into Group-A for control (high-quality embryo rate ≥40%, n=297) and Group-B without high-quality embryo (excellent embryo rate=0, n=427). Meanwhile, based on different effective blastocyst formation rates, Group-B was further divided into two subgroups, Group-B1 with embryonic development potential (effective blastocyst formation rate ≥20%, n=159) and Group-B2 without embryonic development potential (effective blastocyst formation rate=0, n=230).

### Outcome measures:

The factors influencing the development potential of high-quality embryos on D3; and factors influencing blastocyst development potential of late-stage embryos in cycles without high-quality embryos on D3.

### Statistical analysis:

Data analysis in this study adopted SPSS23.0 statistical software. The confidence interval was 95%. χ^2^ test was used for inter-group comparison of counting data, and independent sample t-test for inter-group comparison of measurement data represented by (*χ̅*±*S*). A statistically significant difference was determined when P<0.05. Binary logistic regression analysis was conducted to adjust for confounding factors.

## RESULTS

A total of 724 cycles were involved in this study, of which Group-A had a high-quality embryo rate of ≥40% on D3, and Group-B had a high-quality embryo rate of 0 on D3, including 297 cycles and 189 cycles, respectively. There were no statistically significant differences in male age, years of infertility, body mass index (BMI), baseline FSH levels, AMH, total Gn dose, total days of Gn administration, sperm concentration, motility, normal morphology rate, and LH levels on the day of (hCG injection between Group-A and Group-B (all *p>*0.05). Statistically significant differences were observed in female age, estradiol and progesterone levels on the day of hCG injection, and the number of follicles with a diameter of ≥14 mm between female partners from Group-A and Group-B (all *p*<0.05). Table-I

**Table-I:** Comparison of general data between Group-A and Group-B.

Items	Group-A (n=427)	Group-B (n=297)	p
Female age	31.47±4.22	32.04±3.97	0.032
Male age	32.81±4.98	33.23±4.46	0.244
Female BMI	23.80±3.71	24.15±8.63	0.510
Years of infertility	3.55±2.44	3.69±2.11	0.445
Baseline FSH	6.12±1.97	6.04±3.04	0.610
AMH	3.24±1.93	3.15±2.14	0.545
Total Gn dose	3020.79±1087.55	3078.58±1041.17	0.471
Days of Gn administration	12.69±2.00	12.62±2.05	0.650
E2 on the day of hCG injection	14702.14±8758.90	12375.73±8693.76	0.002
LH on the day of hCG injection	1.14±0.83	1.13±1.02	0.864
P on the day of hCG injection	3.33±2.02	2.99±2.07	0.028
The number of follicles with a diameter of ≥14mm	11.82±4.72	10.65±4.67	0.001
Sperm concentration	71.29±58.57	68.88±72.42	0.634
Sperm motility	33.03±18.79	33.08±31.28	0.983
Normal sperm morphology rate	4.81±2.30	4.97±2.25	0.372
The proportion of primary infertility	199/427	140/297	

In order to reduce confounding factors, factors with p<0.05 in univariate analysis were included in the Logistic regression model for binary regression analysis. Logistic binary regression results revealed no independent risk factors affecting the rate of high-quality embryos in long protocols (Table-II).

**Table II:** Variables in the equation.

Variables in the equation									
		B	Standard error	Wald	Degree of freedom	Significance	Exp (B)	95% confidence interval of EXP(B)	
							Lower limit	Upper limit
Step 1^a^	Female age	-.024	.022	1.165	1	.280	.977	.936	1.019
	E2 on the day of HCG injection	.000	.000	.879	1	.348	1.000	1.000	1.000
	P on the day of HCG injection	.023	.047	.239	1	.625	1.023	.933	1.122
	The number of follicles on the day of HCG injection	.028	.026	1.158	1	.282	1.028	.977	1.082
	Constant	-.217	.777	.078	1	.780	.805		

a. Variables entered in Step 1: Female age; E2 on the day of hCG injection; P on the day of hCG injection; and the number of follicles on the day of hCG injection.

For in-depth analysis of factors affecting the developmental potential of late-stage embryos in cycles without high-quality embryos, Group-B was further divided into two subgroups based on the effective blastocyst rate of freezing, including Group-B1 with embryonic development potential (effective blastocyst formation rate ≥20%, n=159) and Group-B2 without embryonic development potential (effective blastocyst formation rate=0, n=230). There were no statistically significant differences in female age, male age, years of infertility, BMI, baseline FSH, AMH, total Gn dose, total days of Gn administration, sperm concentration, motility, normal morphology rate, LH, estradiol, and the number of follicles with a diameter of ≥14 mm between Group-B1 and Group-B2 (all p>0.05). A statistically significant difference was detected in progesterone level on the day of hCG injection between Group-B1 and Group-B2 (all p<0.05) Table-III. Progesterone level on the day of HCG injection was an independent risk factor affecting the developmental potential of late-stage embryos (Table-IV).

**Table-III:** Comparison of general data between Group-B1 and Group-B2.

Items	Group-B1 (n=230)	Group-B2 (n=159)	p
Female age	32.15±3.90	31.99±4.05	0.698
Male age	33.14±4.65	33.38±4.47	0.603
Female BMI	24.05±3.51	24.38±11.31	0.723
Years of infertility	3.72±2.59	3.78±2.44	0.833
Baseline FSH	6.04±2.30	6.14±2.08	0.654
AMH	3.31±1.93	2.95±2.14	0.115
Total Gn dose	3041.90±1113.39	3140.95±983.02	0.356
Days of Gn administration	12.70±2.29	12.63±1.97	0.387
E2 on the day of hCG injection	11691.54±6909.32	12148.33±9502.11	0.634
LH on the day of HCG injection	1.13±0.87	1.15±1.17	0.844
P on the day of HCG injection	2.63±1.62	3.20±2.31	0.009
The number of follicles with a diameter of ≥14mm	10.60±4.53	10.33±4.80	0.584
Sperm concentration	72.70±92.83	65.49±54.48	0.336
Sperm motility	35.75±44.86	31.66±19.08	0.222
Normal sperm morphology rate	4.85±2.26	5.03±2.27	0.479
The proportion of primary infertility	121/230	65/159	

**Table-IV:** Variables in the equation.

Variables in the equation									
		B	Standard error	Wald	Degree of freedom	Significance	Exp(B)	95% confidence interval of EXP(B)	
							Lower limit	Upper limit
Step 1^a^	E2 on the day of HCG injection	.000	.000	.034	1	.853	1.000	1.000	1.000
	LH on the day of the HCG injection	-.008	.152	.003	1	.960	.992	.736	1.338
	P on the day of HCG injection	-.186	.081	5.344	1	.021	.830	.709	.972
	The number of follicles on the day of the HCG injection	.071	.040	3.070	1	.080	1.073	.992	1.161
	Concentration	.001	.002	.634	1	.426	1.001	.998	1.005
	PR	.005	.005	1.094	1	.296	1.005	.996	1.014
	Normal sperm morphology rate	-.094	.060	2.502	1	.114	.910	.809	1.023
	Constant	-.240	.481	.250	1	.617	.786		

a. Variables entered in Step 1: E2 on the day of HCG injection; LH on the day of HCG injection; P on the day of HCG injection; the number of follicles on the day of HCG injection; concentration; PR; and normal sperm morphology rate.

## DISCUSSION

It was found that the developmental potential of blastocysts would be affected negatively by increased progesterone levels, but not by the quality of male sperms. However, previous data^6^ revealed an impact of sperm DNA on the formation time and morphology of blastocysts. Nevertheless, this study just considered conventional parameters of sperm quality, which should be improved in subsequent studies. High-quality embryo rate on D3 is a concern in the field of assisted reproductive technology. Much attention has been attached to factors affecting embryo quality of long protocols on progesterone levels on the day of HCG injection^7^,^8^, yet with controversial conclusions so far.^9^-^11^ In some studies, elevated progesterone level on the day of HCG injection was reported to be able to affect the quality of oocytes and embryos, leading to reduced rates of high-quality and available embryos in the cycle.^12^,^13^ While some other researchers believed that increased progesterone level on the day of HCG injection has no significant impact on the quality of oocytes and embryos.^14^,^15^ Different results described above may be attributed to the difference in study method, grouping, etc.

Elevated progesterone levels during the late follicular phase of COH may be related to the following reasons. Firstly, there are many mature follicles as exogenous FSH can directly stimulate granulosa cell proliferation. Progesterone is an end product of granulosa cells that cannot be further metabolized, and its level in serum may be elevated when the synthesis of progesterone exceeds the metabolic capacity of the liver.^16^,^17^ The second factor is the Gn dose. It has been reported that under high doses of Gn, FSH stimulates granulosa cells to increase sensitivity to LH, leading to an increase in the conversion of cholesterol into P.^18^

Some other scholars found no statistically significant difference in Gn dose between the elevated progesterone Group-And the normal progesterone group.^19^ Thirdly, it may be related to an excessively high level of LH. In the late follicular phase, LH can synergistically act with FSH on granulosa cells to promote progesterone biosynthesis.^20^ The last factor is the prolonged ovarian stimulation time. In a study carried out by Lawrenz et al.,^21^ prolonged ovarian stimulation time resulted in a significant increase in the rate of elevated progesterone levels.

### Limitations:

However, this study is insufficient to clarify the mechanism completely. Collectively, the above studies analyzed elevated progesterone levels from multiple dimensions, with controversial results generated, which may require further research by including additional data based on more detailed stratification and grouping. In the future, in-depth studies are required to obtain more convincing conclusions based on extended data scope considering potentially biased results due to a smaller sample size in the present study.

## CONCLUSIONS

In conclusion, during the treatment using long protocols, there are no independent factors affecting the quality of embryos on D3. However, for cycles with poor embryo quality, the increase in progesterone levels in the late stage of COH has a significant negative impact on the formation of effective blastocysts.

### Authors’ Contributions:

**HS** and **ZZ:** Carried out the studies, participated in collecting data, drafted the manuscript, and are responsible and accountable for the accuracy or integrity of the work in authors’ contribution.

**QS** and **JL:** Performed the statistical analysis and participated in its design, participated in acquisition, analysis, or interpretation of data and drafted the manuscript.

All authors read and approved the final manuscript.
